# Evaluation of Social Impact Within Primary School Health Promotion: A Systematic Review

**DOI:** 10.1111/josh.13160

**Published:** 2022-04-01

**Authors:** Dianne Robertson, Julia Carins, Sharyn Rundle‐Thiele, Jessica Harris

**Affiliations:** ^1^ Social Marketing @ Griffith Department of Marketing, Griffith University 170 Kessels Road Nathan QLD 4111 Australia; ^2^ Social Marketing @ Griffith Department of Marketing, Griffith University 170 Kessels Road Nathan QLD 4111 Australia; ^3^ Social Marketing @ Griffith Department of Marketing, Griffith University 170 Kessels Road Nathan QLD 4111 Australia; ^4^ Social Marketing @ Griffith Department of Marketing, Griffith University 170 Kessels Road Nathan QLD 4111 Australia

**Keywords:** health promotion, social impact, primary school, evaluation, stakeholder engagement

## Abstract

**BACKGROUND:**

Health promotion programs and interventions are designed to encourage behavioral changes in children, encouraging them to make safe and healthy life choices. This systematic review seeks to examine how social impact is measured in primary school health promotion interventions.

**METHOD:**

A systematic search and review process was used to identify and examine primary school health promotion interventions. The PRISMA guidelines were followed to source articles from 6 electronic databases reporting school health promotion programs or interventions in Australia, Canada, New Zealand, or the United Kingdom.

**RESULTS:**

A total of 77 studies were located, representing 55 health promotion interventions delivered in primary school settings. Of these interventions, only 8 (15%) measured or attempted to measure social impact, whereas another 8 (15%) alluded to social impact. The predominant theories reported were social based theories (theories which examine the social influences on people, environments, and behaviors) (n = 17, 59%), with almost a third not informed by an overt health promotion framework or model (n = 34, 59%). A systematic rating system identified some level of stakeholder engagement (n = 30, 53%).

**CONCLUSIONS:**

This systematic review highlights the need for social impact measurement within health promotion to illuminate the role of school programs in delivering lasting change.

Health promotion was first defined in the *Ottawa Charter* (1986), and subsequently updated in the *Bangkok Charter for Health Promotion in a Globalized World* (2005) and is now defined as “the process of enabling people to increase control over their health and its determinants, and thereby improve their health.”^1^, ^2^ Health promotion includes awareness and knowledge campaigns, health information and advice, and actions which aim to influence broader change at the social, environmental, policy, or economic levels.^3^ Within a school context, health promotion has been defined as a project, program, or initiative which aims to “promote health, health behaviour, health‐related competencies or other social and material determinants of health for students or other school‐related stakeholders.”^4^ ^(p. 196)^ This definition indicates a desire for broader change elements or a focus beyond individual behavior change within school health promotion programs.

Worldwide, a huge array of health promotion interventions have targeted children's health behavior, in response to a range of complex issues. Health behaviors targeted within school‐based contexts include mental health,^5^, ^6^ obesity,^7^, ^8^ nutrition, and physical activity.^9^, ^10^, ^11^ Regardless of the behavior targeted, health promotion in schools is considered important to lay the foundations of healthy living as schools are perceived as the most accessible and consistent platform, with important environmental and social structures to support engagement with children.^12^

Despite definitions of health promotion indicating a desire to create broader change or to have impact at a broader level, the literature on primary school‐based interventions does not show a clear translation into practice. Currently, the majority of the literature reports measuring or evaluating the effect at an individual level or for an individual health determinant, rather than exploring the impact for an individual, or for a broader cohort, community, or population.^13^, ^14^, ^15^ Furthermore, systematic reviews on children's health promotion interventions have synthesized information differently, which can make comparisons challenging when evaluating the theories, intervention durations, intervention components, outcomes and impacts of the health promotion.^16^, ^17^, ^18^ This variation in synthesis of studies reporting health promotion interventions leads to a lack of clarity around the theoretical basis for creating health behavior change; limited capacity to make clear links that attribute any changes observed, or resulting impact, to program elements; and a lack of a consensus about the most appropriate methods for evaluating effectiveness of health promotion interventions.

## Social Impact Resulting from Primary School Health Promotion

Defining impact remains challenging as variation occurs in conceptualization and operational definitions of social impact.^19^ Within the health promotion context, social impact has been defined as the process of analyzing and measuring the economic, social, and environmental consequences of business activity, both the positive and negative, regardless of the purpose or perceived or real benefits of the activity.^20^ Within health promotion, impact is often discussed as an outcome or an effect rather than a benefit. There appear to be 2 main drivers for examining the social impact of health promotions within primary schools. Firstly, there is a growing need for outcome measurement to demonstrate and evidence the impact and value of the health promotion, with increasing pressure for standardization, verifiability, and accountability in meeting delivery and reporting requirements.^21^, ^22^ Secondly, there are calls within the literature for broader measures beyond outputs and short‐term individual measures to support investment in initiatives that deliver lasting behavioral changes within complex systems.^23^

Behavioral change programs that target health issues need to consider broader social, economic, and environmental consequences (both positive and negative) when designing, implementing, and evaluating interventions. This requires consideration of and collaboration with stakeholders to establish 3 key things: *what the impact is*, *who the impact is for*, and *how to evidence impact*.^24^ Consultation with multiple stakeholders in children's health promotion is required to understand the desired behavioral outcomes and objectives, which will guide what should be measured to show if behavioral change has occurred, and to understand the impact beyond individual behavior change following the intervention.^25^, ^26^ Stakeholder engagement may be critical for effective health promotion that aims to achieve social impact.

## Previous Research Reviews

Recent systematic reviews on health promotion have focused on direct, measurable outcomes of interventions such as awareness, knowledge, behavior (either observed or reported, for example, increased consumption of fruit, levels of physical activity, or intentions to not start smoking)^27^, ^28^ or anthropometric measurement (standard body measurements such as such as weight, height, skinfolds, and waist circumference).^29^, ^30^ This focus on direct, measurable individual outcomes is reflected in current reviews on healthy eating (inclusive of nutrition and obesity prevention) which have found that outcomes were predominately anthropometric change, with mixed results reported for efficacy and use of theoretical frameworks.^12^ Physical activity reviews found similar results with a strong focus on measurable outputs and objective measures of moderate to vigorous physical activity (MVPA) levels.^31^ Multiple component interventions, predominately targeting healthy eating, physical activity, and/or healthy living habits, have also focused on individual outcomes such as intentions and behaviors toward health living^32^ and change in anthropometric measures and physical activity levels.^33^ Some previous reviews have considered the role of stakeholders in the process.^13^, ^34^ Although these reviews are important for establishing how programs lead to direct, measurable outcomes, they do not increase understanding of how programs may lead to social impact. Reviews of the social impact arising from school health promotion interventions are lacking.

This systematic review seeks to broaden the current focus of health promotion in primary schools beyond the outcomes that are normally considered (eg, a behavior change or awareness) to understanding the potential or perceived social impacts that are being achieved. To do this, the literature on current health promotion interventions was examined to determine whether the social impact is being considered, and what the current trends are for capturing or measuring social impact in primary school contexts. The purpose of the review is 2‐fold: firstly, to understand how social impact is considered and measured in health promotion; and secondly, to highlight the key learnings for social impact measurement in health promotion for primary school children to guide future health promotion interventions.

## METHOD

Primary school health promotion interventions were sought from 4 Commonwealth countries deemed to have comparable health systems and similar approaches to public health prevention and health promotion in schools. The PRISMA protocol was used to ensure the review was a measurable, reproducible, and comprehensive method to map the relevant literature.^35^ Six databases were searched (EBSCO, Emerald, Ovid, ProQuest, Scopus, and Web of Science) with the following search terms: (“health promotion” OR “health prevention” OR “health program” OR “health initiative” OR “health intervention” OR “health education”) AND (“sex*” OR “drug” OR “alcohol” OR “tobacco” OR “nutrition” OR “obesity” OR “physical activity” OR “fruit*” OR “vegetable*” OR “healthy eating” OR “mental health” OR “wellbeing” OR “well‐being” OR “well being) AND (“primary school” OR “elementary school” OR “primary school‐based” OR “primary school based”).

Records were screened against the following exclusion criteria: (i) papers not in English; (ii) no full text available, (iii) not relevant to health promotion in children, (iv) interventions, initiatives or programs for diagnosed medical or psychological conditions or treatment focused; (iii) countries other than the 4 Commonwealth countries (Australia, Canada, New Zealand, and the United Kingdom); (iv) studies that were not in a primary‐school setting delivered to students; and (v) studies that were conceptual or review papers. Full‐text articles for the retained articles were retrieved, and grouped according to country. Study details were recorded by: (1) author and year of publication, (2) type of theory and/or health promotion framework reported, (3) behavioral focus/intervention approach of the health promotion intervention, (4) sample set used in the evaluation, (5) duration of the intervention, (6) type of evaluation study design and methods used for intervention, (7) the reported outcome effects/results of the intervention, (8) the level of stakeholder engagement in the intervention, and (9) presence and nature of any social impact measurement. Stakeholder engagement was assessed as 1 of 5 levels: (i) inform (informing or educating), (ii) consult (feedback/information), (iii) involve (with consideration and understanding), (iv) collaborate (engaged in a partnership toward plans/actions), and (v) empower (involved in decisions or desired outcome processes)^25^ or “not reported.”

The National Health and Medical Research Council's (NHMRC) quality assessment framework was used to grade the study evaluation design used in each intervention, from I (highest) to IV (lowest) to assess the level of evidence each evaluation's contribution to the evidence base.^36^ Data extraction and assessments were completed by 3 researchers, and when disagreements (n = 4 issues) were encountered, consensus was achieved through discussion. Variation in outcome measures was expected; therefore, meta‐analysis was deemed an inappropriate method of analysis without substantial data transformation and assumptions.

## RESULTS

**Figure 1:**
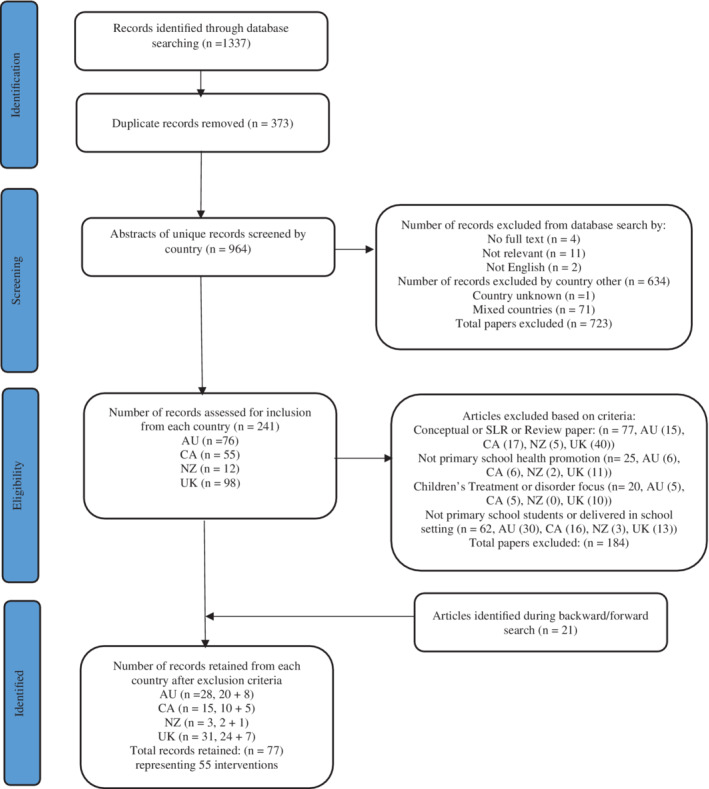
PRISMA Diagram Showing Systematic Search Process

The systematic search retrieved 1333 records. Once duplicates were removed, 964 unique titles and abstracts were screened against the inclusion and exclusion criteria to ensure they were within the scope of the study. After screening, 56 studies remained. Backward/forward searching identified 20 additional records, resulting in a data set of 77 articles representing 55 health promotion interventions. This process is outlined in Figure 1.

These 55 interventions included: 20 Australian interventions, 10 Canadian interventions, 2 New Zealand interventions, and 23 UK interventions. The health promotion interventions commonly focused on healthy eating (n = 29, 52%) and physical activity (n = 25, 45%). They were predominantly focused on a single issue (n = 43, 78%), such as healthy eating (including multiple aspects such as obesity prevention, fruit and vegetable consumption, and reduced intake of sweet drinks), with a small number having multiple foci (n = 13, 24%), such as healthy eating and physical activity. Multiple foci interventions had a broader policy or environmental strategy. Single focused interventions often aimed at preventative or reduction behaviors of harm such as sun safety, sexual health or drug, and alcohol strategies, with the exception of interventions which targeted increasing fruit or vegetable consumption. The NHMRC evidence rating process rated 20 studies at level II (36%), 12 studies at level III‐2 (21%), 7 studies at level III‐3 (13%), and 17 studies at level IV (30%). This represents a body of evidence of sufficient size and quality to be able to guide practice. However, the variation in outcomes measured makes it difficult to draw any conclusions on whether these interventions resulted in social impact. Sample size and duration also demonstrated this same variation. Sample sizes ranged for students (from “not reported” to 4808) and schools (1‐193) making meaningful comparison equally challenging. Most of the evaluation study designs were randomized control trials (RCTs; cluster or groups) (36%), cases studies (30%), or comparative studies (with concurrent controls) (21%), and without concurrent controls (7%). Regardless of study design, most conducted pre‐post/post‐test. Interventions were a mix of process evaluations (13%), process and outcome evaluations (5%), impact evaluations (5%), a mixture of process, outcome or impact evaluations (4%), Research, Effectiveness ‐ Adoption, Implementation and Maintenance (RE‐AIM) evaluations (2%) or a realist evaluation (2%). See Table 1 for details of the included studies.

**Table 1:** Studies Included in the Systematic Literature Review

No.	Name	Authors	Theory	Behavioral focus/intervention approach	Sample set	Duration	Evaluation study, design, and method	Outcome effect	Stakeholder consultation	Social Impact*
1 AU	Play Zone in Primary Schools	Austin, Caperchione^37^	**T**: Not reported **HPF**: Not reported	↑ PA	Schools (n = 7) Students—not reported Age—not reported	12 months	**Case series with pre‐test/post‐test outcomes** **IV** RE‐AIM T1: baseline T2: 1 month during T3: 6 months during T4: 12 months post Qualitative interviews (content analysis) Workshops and preparatory strategies designed to increase the awareness, knowledge, and skills—strategy planning for intervention	Fidelity (adoption of: preparation strategies, playground changes and games, peer led training No measure of PA Participation and adoption rates were ↑ playground changes, playground games decreased at T3, Peer led training lowest uptake	No	Yes
2 AU	Live Life Well@school	Bravo, Folley^38^	**T:** Not reported **HPF**: Health Promoting Schools (HPS)	↑ HE of students and their families ↑ PA ↓ Obesity levels	Schools: (n = 929‐1843) Students: NR Age: 5‐11 years	2012‐2017 (rolling adoption)	**Case series with /post‐test outcomes** **IV** Program adoption of 10 desirable practices: Curriculum (2) Food and physical activity environment (4) Professional development, monitoring (4) Data collection methodology not reported	June 2017 80% of 10 desirable practices adopted. Curriculum had high adoption. Food and physical activity environment (3 out 4 had high adoption when supported by government programs) Professional development, monitoring and reporting had lowest adoption rates	No	No
3 AU	Go for your life!	de Silva‐Sanigorski, Prosser^39^ Honisett, Woolcock^40^	**T:** Theory driven—socio‐ecological framework **HPF**: HPS	↓ Obesity ↑ PA ↑ HE	Schools: 20 G1 (*I* < 12 months) 30 G2 (*I* ≥ 12 months) 20 G3 (member) Students: not reported Age: 5‐12 years	Rolling adoption and varied uptake depending upon if in G1, G2, or G3	**A comparative study with concurrent controls** **III‐2** Mixed method and cross‐sectional study with stratified sample groups The School Environment Questionnaire (SEQ), Child Health Questionnaire, Economic Resource Questionnaire, The Environmental Questionnaires, Lunch Box survey, Policy checklists (2), Open‐ended questions for parents HE and PA	Evaluation protocol design	Yes—primary key organizational stakeholders **(Consult)**	Yes
4 AU	Kids Matter Primary	Dix, Slee^41^ Graetz, Littlefield^42^	**T:** Theory driven**—**social and emotional learning **HPF:** Not reported	Improve MH	Schools: (n = 96) Students: stratified sample of up to 76 students per school (total n not reported) Age: 10 years	2 year implementation (2007/2008)	**Comparative study without concurrent controls—interrupted time series** **III‐3** Questionnaire 4 time points (teachers), 3 time points (parents) Implementation index: fidelity, dosage and quality Measurement of academic performance (NAPLAN) and teacher perceived performance	Significant + relationship between implementation and academic performance (↑ between 2.6 and 6.2 months)	Yes—primary key organizational stakeholders **(Collaborate)**	Yes
5 AU	SunSmart Policy Intervention	Dudley, Winslade^16^ Dudley, Cotton^43^	**T:** Theory driven—social cognitive theory (SCT) **HPF**: HPS	↑ Wearing sun protective headwear during breaks ↑ SunSmart behavior, ↑ SunSmart Education ↑ SunSmart Policy	Schools (n = 20, IG 5 and CG 15) Students: Grade 5 ‐ 6 Age: G5 and G6	18 months	**Cluster RCT** **II** **T1:** Baseline measurements. Group **T2:** Focus Groups, Interviews Direct observations **T3:** Post‐test—12 months **T4:** Follow‐up—15 months SCT outcomes measured factors: personal, behavioral and environmental	Evaluation protocol design Follow‐up: cross‐sectional design 60% of children wore a sun‐safe hat during their breaks NS increase in sunscreen consumption or other SunSmart behaviors	Yes—primary key organizational stakeholders **(Involve)**	No
6 AU	Fit‐4‐fun	Eather, Morgan^44^ Eather, Morgan^45^ Eather, Morgan^46^	**T:** Theory driven—social cognitive theory (SCT), Harter's competence motivation theory (CMT) **HPF:** HPS	↑ PA ↑ School environment to support PA ↑ Self‐efficacy, social support and motivation	Schools (n = 4, 2 IG and 2 CG) Students (n = 226, IG 118, and CG 108)	60 minutes × 8 weeks during HPE 8‐week home (3 × 20 minutes per week)	**Group RCT** **II** Student questionnaire **T1:** Baseline **T2:** Follow‐up—3 months **T3:** Follow‐up—6 months	T1: Baseline NS difference between groups T2: Significant ↑ perception of school environment T3: Significant difference found between groups, except peer social support and parents. Social support by teachers mediated effect of IG on PA	No	No
7 AU	Stephanie Alexander Kitchen Garden Program (SAKGNP)	Eckermann, Dawber^47^ Gibbs, Staiger^48^	**T:** Theory driven**—**socio‐ecological approach **HPF:** HPS	F&V Eating Habits	Schools: (n = 42, IG 28 and CG 14) Students:(IG 491 and CG 260) Age: G3‐G6	2 years (45 minutes × weekly lessons, 90 minutes kitchen)	**Comparative study with concurrent controls—case‐control study** **III‐2** Grade 6 students surveyed and parent questionnaire pre/post: garden and kitchen lifestyle behaviors, eating habits, food choices	Short‐term impacts ↑ kitchen but ns difference in garden behavior between IG and CG groups 77.4% parents indicated that their children asked for the same foods from the program 71.9% of parents reported their child was more willing to cook at home Significant increases in kitchen lifestyle behaviors IG compared to CG Significant increases in willingness to try new foods in IG compared to CG ↑ Food choices of IG and kitchen lifestyle behavior post 2 years NS difference in eating habits of IG and CG post 2 years Long‐term impacts; SROI as ($226,737/$44,758)	Yes **(Involve)**	Yes
8 AU	School‐based intervention for increasing physical activity	Engelen, Bundy^49^ Grunseit, Hara^50^	**T:** Not reported **HPF:** Not reported	↑ PA	Schools: (n = 12, IG 6 and CG 6) Students: (n = 221, IG 113 and GC 108 C) Age: 5‐7 years	13 weeks 2008/2009	**Cluster RCT** **II** Playground environmental changes and equipment Adult 2‐hour discussion of free play beliefs and value **T1**: Baseline **T2**: Post‐test PA measured by accelerometers, BMI (kg/m^2^), play area per child **T3**: 2 year follow‐up (school n = 1, students n = 16)	Small but significant ↑ PA by I cf. C by more than 12% ↑ in C sedentary time, ↓in I (T3): Small significant ↑ PA maintained and equipment in use Additional measures besides Accelerators require to measure PA	No	No
9 AU	Crunch & Sip Free fruit pilot	Hector, Edwards^51^	**T:** Not reported **HPF**: Not reported	↑ F&V intake	Schools: (n = 4) Students: (n = not reported) Individual classes (n = 55) Age: K‐G6	10 weeks Terms 3 and 42,014	**Comparative study without a parallel control group** **III‐3** Time series, mixed‐methods study design **T1:** Baseline **T2**: Week 9	Significant ↑participation rates in Crunch and sip from baseline to week 9 (46.7% to 92%), with OR 17.5 increase in participating Small increase in bringing FV from home 46.7% to 54%	No	No
10 AU	Traditional Indigenous Games (TIG)	Kiran and Knights^52^	**T:** Not reported **HPF**: Not reported	↑ PA and ↑ cultural connectedness	Schools (n = 4, 2 x 2) Students: (n = 167, IG 91 CG 76,) Age: G5‐G6	Every week for 12 weeks, 2007	**Cluster RCT** **II** Questionnaire **T1:** Baseline **T2:** Follow‐up 1 week post‐intervention	NS ↑ PA and ↑ cultural connectedness or between indigenous or non‐indigenous students	Yes—indigenous reference group **(Involve)**	No
11 AU	Fresh Kids	Laurence, Peterken^53^	**T:** Not reported **HPF:** HPS	↑ Fruit and water intake ↓ Sweet drink intake	Schools: (n = 4) Students: (n = varied upon school, and timeframe) Age: Not reported	2 years (4 schools) 3 years (2 schools)	**Comparative study without concurrent controls** **Interrupted time series without a parallel control group** **III‐3** Impact evaluation **T1**: Baseline Lunchbox observational audit **T2**: 12 months (4 schools) **T3**: 24 months (4 schools) **T4:** 36 months (2 schools)	Significant ↑ at of all schools at T3 fruit intake of 25‐50%, ↑water intake of 15‐60%, ↓ sweet drinks of 8‐38%. T4: Sweet drinks not measured, Intake water and fruit intake remained ↑	Yes—school staff **(Collaborate)**	No
12 AU	Supporting children's outcomes using rewards, exercise and skills (SCORES)	Lubans, Plotnikoff^54^ Cohen, Morgan^55^	**T**: Theory driven—socio‐ecological model (SEM) Self‐determination theory (SDT) Competence motivation theory (CMT) **HPF:** not reported	↑ PA and Fundamental movement skills (FMS)	Schools: (n = 8, IG 4 and CG 4) Students: (n = 460, IG 199 and CG 261) Age: G3‐G4	12 months, 2012	**Group RCT** **II** Process evaluation Matched pair 3 phases: (1) professional development and equipment; (2) policy changes; (3) strategies employed *Questionnaires* Physical activity: Accelerometers (7 days, Cardio‐respiratory fitness (CRF), FMS assessed by Test of Gross Motor Development (TGMD) II, BMI (kg/m^2^) *Psychological measures*: Harter's Self‐Perception Profile (SPP), Child and Youth Resilience Measure (CYRM‐28), Physical Activity Enjoyment Scale (PACES), Children's Leisure Activities Study Survey (CLASS) **T1:** Baseline, Term 1 **T2:** Mid, Term 3 **T3**: Post, Term 1, 2013 (12 months)	Evaluation protocol design Significant treatment effects for locomotor skills and overall FMS FMS competency not perceived competency mediated the effect on PA and cardio fitness	No	No
13 AU	Nutrition Education and Garden (NE&G)	Morgan, Warren^56^	**T:** Theory driven ‐ Social Cognitive Theory **HPF:** HPS	If NE&G ↑ fruit and vegetable consumption and knowledge	Schools: (n = 2) Students: (n = 1G 27, I1 NE 35, I2 NE and G35, CG 57) Age: G5‐G6	10‐week intervention	**A comparative study with concurrent controls** **III‐2** Vegetable intake—24‐hour food recall (baseline and post), Vegetable preference (taste and rate method) Vegetable knowledge (6 vegetables observation and F&V questionnaire adapted from “Gimme 5”—8 questions Quality of school life (QoSL) 3X1 1‐hour NE lessons in the classroom delivered by teachers **T1**: Baseline **T2:** Post‐4 month	NS difference between groups for F&V intake ↑ Significant willingness for NE&G to taste and rate No effect on F&V intake Knowledge increased—able to identify vegetables (knowledge is a construct of SCT)	No	No
14 AU	In‐class vegetable promotion program	Myers, Wright^57^	**T:** Not reported **HPF:** Not reported	↑ Vegetable intake ↑ Teacher perceived nutritional knowledge	Schools: (n = 21) Primary school teachers: (n = 35) Students: (n = 818) Age: 4‐11 years	10‐week, terms 2‐3	**Case Series with** **quantitative pre‐post outcomes** **IV** Mixed methods design with a process and outcome evaluation Part of the Crunch & Sip program **T1**: Baseline survey 8 × nutritional lessons 1‐week vegetable challenge **T2**: End of intervention survey	Significant ↑of vegetables (21% *cf* 46%) Significant ↑in teachers SR perceived nutritional knowledge Teachers attitudes and confidence ↑	Yes—educational staff **(Involve)**	No
15 AU	Physically Active Children in Education (PACE)	Nathan, Wiggers^58^	**T:** Theory driven ‐ Behavior Change Wheel (BCW) and Theoretical Domains Framework (TDF) **HPF:** Not reported	↑ PA	Schools: (n = 62, randomized IG or CG) Students: (n = unknown) Age: G2‐G3 subset	12 months	**Cluster RCT** **II** Cost effectiveness analysis (CEA) **T1:** Baseline **T2:** post‐12 months **T3**: post ‐ 18 months PA daily log‐book for 1 week T1‐T3, accelerometers ≥3 days Questionnaire T1‐T3	Evaluation protocol design	Multiple stakeholders **(Involve)**	No
16 AU	Great Leaders Active Students (GLASS)	Nathan, Sutherland^59^	**T:** Theory driven ‐ Transformational Leadership Theory **HPF:** Not reported	↑ PA and ↑ object control skill	School: (n = 2) Students: (n = 174, IG 83 and CG 91) Age: Gr K‐2	10 weeks, 2015	**A comparative study with concurrent controls—non‐randomized control trial** **III‐2** Blinded group measurement Peer leaders delivered 2 × 30 min object control sessions (catch, underarm and overarm throw) **T1:** Baseline and **T3**: post 3 months PA measured by pedometer for 5 days, Object control skills assessing using Test of Gross Motor Development‐3 (TGMD‐3), participants were videoed performing skills and rated correct or incorrect Peer leadership skills assessed by Transformational Teaching Questionnaire (TTQ)	Ns effect on ↑PA S effect on ↑ object control skill and teacher rated ↑ peer leadership	No	No
17 AU	It made me feel Brazilian	Radicchi, Thompson^60^	**T:** Not reported **HPF:** Not reported	↑ Social inclusion and ↑mental health	Schools: (n = 1) Students: (n = 31) Age: 11‐12 years G5‐G6	5 classes × 60‐minute sessions, weekly Term 4, 2017	**Case series with post‐test outcomes** **IV** Observations Daily field diary Semi‐structured student feedback to 4 responses	Observations and feedback not connected to social inclusion or mental health	No	No
18 AU	Kids in the Kitchen	Ritchie, O'Hara^61^	**T:** Not reported **HPF**: HPS	↑ F&V intake	Schools: (n = 1) Students: (n = 118) Age: Grade 1 and 5	10 weeks, 2007	**Case series with pre‐post‐test outcomes** **IV** Impact Evaluation Questionnaire **T1:** Baseline and **T3**: Post‐intervention: on knowledge, attitudes and F&V consumption. F&V preparation skills and environment, identification and rating of 40 F&V (adapted from Tooty Fruit Vegie project)	Significant ↑ of identification and engagement with F&V. NS change pre‐post in knowledge, attitudinal and consumption statements Decrease in their skills for knife and fork use Cutting, grating and peeling skills increased No change in environmental supports.	No	No
19 AU	Aussie Optimized Program (AOP)	Roberts, Williams^62^	**T:** Not reported **HPF**: Not reported	↓ T&A use	Schools: (n = 62, IG1 AOP + T 20, IG2 AOP + T&C 22, CG 21) Students; (n = 2023, IG1 AOP + T 736, IG2, AOP + T&C 693, CG 594) Age: 10‐13 years 6‐7 grades	10 (SLS) × 60 minutes weekly and (OTS) IG1 and IG2 AOP + T&C 4 hours coaching per student years 1 and 2 over 2 years	**Cluster RCT** **II** AOP Questionnaire **T1:** Baseline at beginning of grade 6 Invention contained: Social skills, social problem solving, challenging unhelpful thoughts. **T2**: post‐test end of grade 7 **T3:** Follow‐up end of Grade 8 Questionnaires for students and parents (T1‐T3)	At T1 higher use of A than T At T3 C students were 1.6 times more likely I2 AOP + T&C. At T2 1.4 times and T3 1.2 times more likely to use alcohol An intervention effect was found for teacher led AOP + coaching, IG students were less likely to smoke or consume alcohol than the CG who were 1.6 times more likely to smoke and 1.2 times more likely at follow‐up (T3)	No	No
20 AU	School‐Based Food Garden	Somerset and Markwell^63^	**T:** Not reported **HPF:** Not reported	↑ Ability to identify F&V ↑ positive attitudes toward F&V	Schools: (n = 1) Students: (n = 152, IG 120 and CG 132) Age: G4‐G7	11 hour/week for 12 months	**A comparative study without concurrent controls—historical control study** **IV** Determinant questionnaire: attitude, self‐efficacy, liking, preferences, knowledge and perceived barriers, social environment F&V identification questionnaire **T1:** Year 1 pre‐school garden T2: Year 2 post‐school garden	↑ Ability to identify individual F&V ↑ Cconfidence in preparing F&V Attitude there was ↓interest in trying new fruits NS difference between IG & CG groups	No	No
21 CA	“Little Cooks”	Bisset, Daniel^64^ Bisset and Potvin^65^	**T:** Theory driven—social innovation model Actor‐network theory **HPF**: WHO Ottawa Charter	↑ HE and nutritional education and experiences	Schools (n = 7) Students: (IG 209 and CG 179) Age: G5 and G6	8 × 90 minutes workshops	**A comparative study without concurrent controls** **III‐3** Nutritional Questionnaire: Knowledge, attitude. Capacity and experience, Parental/family participation in schools Peer‐led	↑ Knowledge of nutritional knowledge and cooking NS difference found in food guide, food produce or international cuisine. Family and/or parental participation and gender (girls higher than boys) were significant covariates	Yes (community, professional and educational staff) **(Collaborate)**	No
22 CA	Healthy Buddies	Campbell, Barnum^66^ Ronsley, Lee^67^	**T:** Not reported **HPF:** Whole‐school health promotion program	↑ Knowledge and attitudes toward (PA) “Go Move!”; eating healthy foods (N); Go Fuel! Healthy body image (HL) “Go Feel Good!	Schools (n = 6) Students: K‐3 (n = 557, IG 364, C 193) 4‐7 (n = 723, IG 509, C 214) Age: K‐3 and 4‐7 grades	21 × 30‐minute sessions, 6 × 30‐minute fitness loops	**Case series (pre/post‐test outcomes)** **IV** *Questionnaire* **T1**: Baseline ‐beginning of school year **T2:** Post‐Questionnaire Children's Eating Attitudes Test (CEAT)—end of school year Healthy living knowledge, behavior, habits and attitudes *Physical measurements*: included weight, height, waist size, BMI, BP and HR	K‐3 and 4‐7 IG significant ↑ HL knowledge than CG. K‐3 IG significant ↑HL and habit scores 4‐7 IG ↑ in all 5 CEAT questions cf. 2 in CG Physical measures ↑ age	Yes Indigenous communities Peer‐led **(Involve)**	No
23 CA	Action Schools! BE—healthy eating (AS! BC—HE)	Day, Strange^68^ Naylor, Macdonald^69^	**T:** Theory driven ‐ SEM **HPF:** whole school framework	↑ F&V intake	Schools: (n = 10, IG and CG 5) Students: (IG 246 and CG 198) Age: Gr JK‐8	2 × HE activities per week (12 weeks)	**Non‐randomized experimental trial with control** **III‐2** Process Evaluation Fruit intake Vegetable intake Student's knowledge, attitudes and perceptions of FV, ↑ Willingness to try new FV recall, Fidelity to Classroom dose, Food Frequency Questionnaire (FFQ), 24‐hour Food Recall	↑ in F, FV servings, FV variety and percentage of FV tried in the intervention schools. Teachers implemented activities across 80% of whole‐school No change in knowledge, attitudes and perceptions No change in willingness to try new FV	Yes Ministry of health, the MTSA, the Ministry of Education, 2010 Legacies Now, Provincial Health Services Authority Advisory committee (PAC), key public health, recreation, and sport stakeholders **(Collaborate)**	Yes
24 CA	Northern Fruit and Vegetable Pilot Program (NFVPP)	He, Beynon^70^	**T:** Not theory driven—social cognitive theory (SCT) **HPF**: not reported	↑ F&V intake, ↑ nutritional knowledge	Schools IG 1 FFVS+ENE 9, IG2 FFVS 9, CG 8 Students: (n = 1277, IG1 FFVS+ENE 400, FFVS‐alone 470, CG 407 Age: G JK‐8	21 weeks	**Cluster RCT** **II** Impact Evaluation **T1:** Baseline **T2:** End of intervention 24 hour F&V recall questionnaire (servings/d) *Psychosocial & behavioral measures*: Pro Children Questionnaire	IG1 (0.49 serving/day) and IG2 (0.42 servings/day), consumed more F&V than CG, however, only IG1 was significant ↑ variety of F&V reported in IG1 and IG2 NS difference between groups for psychosocial and behavioral scales	No	No
25 CA	Kahnawake Schools Diabetes Prevention Project	Macaulay, Paradis^71^ Adams, Receveur^72^	**T:** Theory driven ‐ Behavior change Theory, Native Learning Styles, Social Learning theory, Precede‐Proceed **HPF:** OCHP, Health promotion planning model	↓ (NIDDM) in a native community Short term: ↓Obesity, high calorie/fat diets, ↑PA and HL	Schools: (n = 2) Students (n = 458) Age: G1–G6	10 × 45 minute lessons per year for each grade	**Case series with Post‐test** **IV** Mixed longitudinal and Cross‐sectional design Outcome, proximal impact, and process evaluation *Physical measures*: Fitness run/walk test Body composition (weight, height, skinfold thickness *Behavioral*: Eating habits (7‐day food frequency questionnaire) Physical activity patterns (questionnaire)	63 Interventions were delivered in school and community. Anthropometric data ↑ with age, ↓ fitness and ↑ television screen time for students ≥9 years	Yes Indigenous Population Community consultation **(Empower)**	No
26 CA	Passport: Skills for Life (PSL)	Mishara and Dufour^73^	**T:** Not reported **HPF**: Not reported	↑ Children's coping skills ↑ Good (MH)	Schools: (n = 20, IG 12, CG 8) Students: 9, n = 1492, IG 666 and CG 826 Age: G3‐G6	1 Intro session and 17 × 55 minutes sessions	**RCT** **II** **T1:** Pre‐test **T2**: post test **T3:** follow‐up (12 months) Teachers' questionnaire Observations of 89 sessions Focus groups after program *Quantitative measures*: Emotional awareness LEAS‐C Coping (Coping in hypothetical situations, draw and write, Kidscope, Children's Coping Questionnaire (CCQ) Social and Academic skills: Social Skills Rating (SSRS) Draw and write	Small significant ↑ coping skills and strategies of IG cf. CG, maintained at post‐test. NS difference between T1 and T2, but significant difference between T1 and T3 for CCQ measure of coping between IG and CG. NS difference between T1 and T2, but significant difference between T1 and T3 for social and academic skills between IG and CG. Focus groups increased appreciation (exceeded my expectations')	Yes—students and teachers in program development **(Involve)**	No
27 CA	Zippy's Friends	Monkeviciene, Mishara^74^ Dufour, Denocourt^5^	**T:** Theory driven—coping model **HPF:** Not reported	↑ Coping skills	School: not reported Students: (n = 246, IG 140 and CG 106) Age: G1	24 weekly sessions	**Comparative Study with concurrent controls:** **III‐2** *Questionnaires*: The behavioral and Emotional Adaption to the Transition, the Problems Encountered, the reactions Observed in the New School Environment	IG had significant ↑ behavioral and emotional adaptions to school than CG. IG had significant ↑ positive reactions to new school and ↑coping skills and strategies than CG. Evaluation found significant differences for IG cf. CG with ↓ Internalization, ↑ co246operation, autonomy and perceived social support.	No	No
28 CA	Prince Edward Island—school nutrition policy (PEI SNP)	Mullally, Taylor^75^	**T:** Not reported **HPF:** Not reported	↑ HE through nutrition school policies (NSP) and ↑ F&V intake, ↑ milk and alternative intake, ↓ LNDF	Schools: (n = 2, 1G1 (2007), CG (2001/2002) Students: IG 562, CG 917 Ages: G5‐G6	5‐year period	**Comparative study with non‐concurrent controls** **III‐3** Quasi‐experimental design **T1**: Pre‐NSP policy survey 2001/2002 **T2**: Post‐NSP policy survey 2007 The Eating Behavior Study (EBS), Food consumption food frequency questionnaire No measure of NSP changes or food environment	Intervention students were 2.14 more likely to have less than 3 daily serves of LNDF, and 1.44 more likely to meet F&V recommendations and 1.27 more likely to choose MA than comparison students.	No	No
29 CA	APPLE Schools	Ofosu, Ekwaru^76^	**T:** Not reported **HPF:** OCHP CSH	↑ HE ↑ PA ↑ Good mental health (MH)	Schools: (n = 26, IG 13, CG 13) Students: (n = 540) Age: *M* = 13.8 ± 1.4 of IG, 14.0 ± 1.3 CG	2015/2016	**Comparative study without concurrent control—historical control study** **III‐3** *Post‐survey*: Youth Health Survey (YHS) (students) Home survey (parents) Survey on: Knowledge, Attitudes, Self‐efficacy and diet *Physical measures*: Physical activity (pedometer) over 9 consecutive days Weight and height BMI (kg/m^2^) Parent and student demographic *Dietary intake*: 24 hour dietary recall WEB_Q 24)	NS difference between APPLE and Comparison schools on outcomes NS difference between historical comparative and current study	Parents, community and “other stakeholders” **(Collaborate)**	Yes
30 CA	Choices and Changes	Wackett and Evans^77^	**T:** Not reported **HPF:** CGSHE	Sexual Health Education (SH)	Schools: (n = 1) Students: (n = varied depending upon measure and (T): Age: G4‐G7	3 years (1998‐2001) Grades 4‐6 (8 × 1‐hour session, 2 per week for 4 weeks) Grade 7 (9 × 1)	**Case Series (pre‐post‐test outcomes)** **IV** Questionnaire **T1:** pre **T2**: end **T3**: post 1 month **T4**: post 3‐4 months Knowledge acquisition, motivation and personal insight, assertiveness skills, supportive environments	Significance not assessed—descriptive measures only ↑ Knowledge, motivation and personal insight maintained at T4	Yes (parents/guardians input into program objectives →parent component added) **(Consult)**	No
31 NZ	Pilot‐Free Fruit—Auckland	Ashfield‐Watt, Stewart^78^	**T:** Not reported **HPF:** Not reported	↑ Fruit intake Provision of free fruit	Schools: (n = 20) Students: (n = 2032, IG 1035 and CG 997) Only 490 completed (T1), (T2), (T3) Age: 7‐11 years (Low SES, Pacific and Maori 81% of group)	10 weeks Term 1	**Paired, Clustered Randomized Control (RCT)** **II** **T1:** Pre 1 week prior **T2**: During week 10 **T3:** Post 6 weeks Day in the Life Questionnaire (DILQ)	↑ Fruit intake IG (0.4 pieces/school d). Reduced consuming no fruit by 22% ↓ but decreased after T3 At T1 NS difference between CG cf. IG 19% IG group ate fruit at T1 and T2 T2's ↑ fruit intake IG cf. CG T3 ↓ fruit intake of IG cf. CG At T2 9% had ↑ fruit intake overall, 32% ↑ T2 but ↓fruit intake at T3, 5% maintained fruit intake from T1 to T3, and 13% reported 0% fruit intake	Yes Maori and Pacific peoples' Representatives **(Consult)**	No
32 NZ	Project Energize	Rush, McLennan^79^ Rush, Cairncross^80^	**T:** Not reported **HPF:** Whole‐school approach	↑ HE ↑ PA ↑ physical fitness ↓in the overweight and obesity rates ↓ in Type 2 diabetes	Schools (n = 193) Children n = 4808 Age: 6‐11 years Population sample characteristics	2009‐2011	**Randomized Control Trial** **(RCT)** **II** 2004 and 2006 control Intervention children for 2011 *Physical Measures*: BMI (h/kg^2^), International Obesity Task for—Obesity Physical Fitness—550 M run (Time) Health, knowledge, and behaviors measured Program effectiveness	↑ Physical fitness HE not measured Overall, BMI, overweight/obesity levels were less than historical comparison ↑ Speed (approx. 10% than historical comparisons) Ongoing evaluation effective ↓ obesity, ↑ physical fitness) and cost effective and efficient ($45/child/year)	No	No
33 UK	WAVES	Adab, Barrett^7^ Clarke, Griffin^81^	**T:** Not reported **HPF:** Not reported	↑ PA	Schools: (n = 54, 26 IG and 28 CG) Students: (n = 1387) Age: 6‐7 years Schools: (n = 10) Parents: (n = 30) Students: (n = 62) Age: 6‐7 years	12‐month program	**Cluster RCT** **II** Process Evaluation QALYS Cooking workshop Signposting of PA opportunities PA component Villa Vitality 24 hr dietary intake *Physical Measures*: BMI (h/kg^2^), waist circumference, skinfold thickness, body fat percentage **Qualitative Study** Focus groups were held with 30 parents and 62 children	Overall there was NS difference between IG and CG Initiate positive behavior changes in families, and indicated that a combination of pathways: knowledge and skills of children and parents; parental empowerment and role modeling; opportunities to lead healthier lifestyles	Yes **(Involve)**	No
34 UK	Marathon Kids	Chalkley, Routen^82^ Chalkley, Routen^83^ Chalkley, Routen^84^	**T**: Not reported **HPF**: Not reported	↑PA Identify the contextual factors ↑ implementation effectiveness Identify the processes of implementation ↑PA Students are able to complete a marathon distance over a year	Schools: (n = 6) Students: To be confirmed Age: G4 Schools: (n = 5) Students: (n = 9) Age: years 6‐10	1‐2 week over 2016 year during lunch breaks ‐2 weeks over 2016/2017 during lunch breaks	**Case series with pre‐post‐test outcomes** **IV** Evaluation protocol design Realist Evaluation, Mixed Methods (T1 and T6) Semi‐structured Interviews, demographics, anthropometrics and PA, (T2, T5, T6, and T8) teacher interviews, pupil focus groups, observations, T3, T4, Radio frequency IDs, weekly teacher log and participation data *School environment*: Sport, Physical activity and Eating Behavior, Environmental Determinants in Young People (SPEEDY), International Study of Childhood Obesity, Lifestyle and the Environment (ISCOLE), Questionnaire Assessing School Physical Activity Environment (Q‐SPACE‐R) Physical Activity Questionnaire for Children, International Fitness Scale *Psychosocial measures*: Self‐efficacy, enjoyment, Global Self‐worth, athletic competence, attitude, social support, intention and motivation Case series with post‐test outcomes IV Qualitative semi‐structured focus groups Observations Questionnaires Teacher implementation logs	Distance = laps completed with lap bands All schools implemented with good fidelity, level of implementation varied Average distance per pupil per week ranging from 0.02 to 2.91 km and boys ↑ participants cf. girls Students found MK ↑ PA Peer influence on participation was important with both positive and negative influences on social cohesion and competitiveness Goal setting and rewards were seen as important Teacher influence was important on student engagement levels	Yes **(Consult)** **Yes—at process level (Collaborate)**	No No
										
35 UK	Zippy's Friends	Clarke, Sixsmith^85^	**T:** Theory driven—coping framework **HPF**: not reported	↑MH ↑EW Participatory approach to understanding participants perspective	Schools: (n = 44) Students: 9 (n = 161, IG 84, CG 77) Age: not reported	24 weeks	**Cluster RCT** **II** **T1:** Pre **T2:** Interim **T3:** Post T1 and T3 Draw and write technique Emotional literacy (T1 and T3) Brainstorming (T3)	4 themes: conflict, rejection, loss and injury IG had a broader range of vocabulary and understanding in relation to emotions concerning problem situations Positive impact on problem solving and support‐seeking strategies	Yes **(Collaborate)**	No
36 UK	“Project Spraoi”	Coppinger, Lacey^86^ O'Leary, Rush^87^	**T:**Theory driven**—**SEM of Health Behavior **HPF**: Not reported	↑ PA ↓ Obesity levels ↓ Sedentary lifestyles ↑ Eating habits	Schools: (year 1, n = 6, IG 4 and CG 2; year 2 (additional), n = 10, IG 3 and CG 1; year 3 (proposed), n = 15, IG 3 and CG 2) Students: Not reported Age: 6‐10 years	3 years	**RCT** **II** RE‐AIM **T1:** Pre **T2**: Post **T3:** Follow‐up *Physical measures*: Body Fat, BMI, BP NE and resources for students and parents	To be completed ↑reach Effectiveness for 10 years ↓ waist size and heart rate Mixed results for Nutritional education between IG and CG for 6 and 10 years NS changes in BMI	Yes—community consultation and consideration **(Consult)**	No
37 UK	Project Tomato	Evans, Ransley^88^	**T:** Theory driven—health maintenance behaviors **HPF:** not reported	If F&V intake can be maintained post free F&V	Schools: (n = 52, IG 26 and CG 24) Students: (n = 658, IG 311 and CG 347) Age: 7‐8 years, G2	10 months IG—12 lessons, 2 newsletters, parents' advice, take‐home activity bags CG received “5‐A‐DAY” booklet and healthy eating leaflets only	**Cluster RCT** **II** **T1:** Baseline **T2:** Follow‐up—20 months after baseline Questionnaire on F&V consumption *Food intake*: 24‐hour dietary assessment recall	NS difference between groups ↓ F&V both groups (T2) ↓ Implementation of intervention	No	No
38 UK	Love Life *Smokefree Sports* Program	Fairbrother, Curtis^89^	**T:** Not theory driven **HPF:** Not reported	↓T uptake	Schools: (n = 2) Students: (n = 120) Age: 10‐11 years	8 weekly × 30‐60‐minute sessions	**Case series with post‐test outcomes** **IV** Qualitative focus group—thematic analysis	Health messages (not measured) Knowledge awareness (not measured) Link between activities and health messages ↓ recall	No	No
39 UK	Children's Health, Activity and Nutrition: Get Educated! (CHANGE!)	Fairclough, Hackett^90^	**T:** Not theory driven—social cognitive theory **HPF:** not reported	Promote healthy weight by ↑ PA and HE “move more, sit less” ↑ Nutrition knowledge	Schools; (n = 11, IG 5 and CG 6) Students: n = 318, IG 89 and CG 117 Age: 10‐11 years	2010/2011 20 × 60 min weekly lesson	**Cluster RCT** **II** **TI:** Baseline **T2**: post 20 weeks **T3**: follow‐up—30 weeks *Physical measures*: waist size, BMI (kg/m^2^), accelerometers for PA 7 days, playground area *Food intake*: 24‐hour recall questionnaire	Significant ↓waist size at T2‐1.07 cm BMI ↑ T1 → T2 for IG and CG, but significant ↓T3 for IG ↑ Light PA ↑ IG students in playground area than CG Intervention effects found to be most effective for overweight/obese students, and ↑SES families	Yes, parents' children and teachers **(Involve)**	No
40 UK	Citizenship Safety Program (CSP)	Frederick and Barlow^91^	**T:** Not theory driven Social Learning Theory, Diffusion of Innovation and Social Inoculation Theory **HPF:** Not reported	Accident Prevention and ↑ risk awareness	Schools: (n = 2, 1 primary and 1 secondary [peer]) Students: (n = 76, 54) Age: G2 (6‐7 years) and 10 (14‐15 years)	30 minutes × 10 weekly sessions	**Case series with pre‐post‐test outcomes** **IV** Process and outcome evaluation **T1**: Baseline **T2:** End **T3**: post 2 months *Measures*: Draw and write on safety topic at T1‐T3, diaries, interviews with teachers T2 Pictorial survey T2, year 10 Peer led tutoring for year 2 students on accident prevention and risk awareness	T1‐T2 had a reported ↑ accident prevention and risk awareness. T3 outcomes not reported	Yes—formative design **(Consult)**	No
41 UK	Nutrition Education at Primary School (NEAPS)	Friel, Kelleher^92^	**T**: Theory driven—social learning theory **HPF:** HPS	↑ HE and HE behaviors, ↑ nutrition knowledge	Schools: (n = 13, IG 10 and CG 3) Students: (n = 821, IG 453 and CG 36) Age: 8‐10 years	20 × 30 minutes sessions over 10 weeks	**A comparative study with concurrent controls—non‐randomized trial** **III‐2** **T1**: Baseline **T2**: Post *Measures*: Food diaries and validated food pairing questionnaire on food behavior, knowledge completed at T1 and T2	NS ↑ in nutrition knowledge IG ↑ F&V intake and ↓ salty snacks at T2 ↑ in children's behavior	No	No
42 UK	The Lifeskills Program	Gabhainn and Kelleher^93^	**T:** Not reported **HPF:** HPS	↑ Health promoting behavior	Schools: (n = 33 post‐primary) Students: post primary (PP) (n = 1620 with 795, Lifeskills− and 825 Lifeskills+), Young adults (YA) (n = 317, 129 Lifeskills− and 188 Lifeskills+), Age: PP 12‐17 years, YA 18‐25	Not reported	**A comparative study with concurrent controls—interrupted time series with a control group** **III‐2** *Measures*: Health Behavior in Schoolchildren (HBSC) Questionnaire, Rosenberg Self‐Esteem Scale, The General Well Being Questionnaire, The Children's Locus of Control Scale, The Multidimensional Health Locus of Control, The Mastery Scale, Lifeskills knowledge questionnaire	Main impact is a significant ↓ alcohol intake Lifeskills+ group All other significant effect was found on health behaviors, for example, smoking	No	No
43 UK	WAVES study	Griffin, Clarke^94^	**T:** Not reported **HPF:** Not reported	Obesity prevention program ↑PA, ↑HE skills	Schools: (n = 24), Students: Not reported Age: 6‐7 years, G2	Villa Vitality 6‐week program	**Cluster RCT** **II** Process Evaluation *Measures*: observations, questionnaire, logbooks, teacher and student focus groups and teacher interviews Fidelity to PA, Cooking workshops and Villa Vitality (healthy lifestyle program)	Schools implementation fidelity: 8 had low, 8 had medium, and 8 had high. Lowest area of fidelity was PA	Yes—families in formative stage **(Consult)**	No
44 UK	The Daily Mile	Harris, Milnes^95^	**T:** Not theory driven—theory of change **HPF**: not reported	Understand the implementation factors, impact and context which affect ↑ PA in the Daily Mile at individual, school and community level	Schools: (n = 1) Students: (n = 75) Phase 1 (n = 75), Phase 2 (n = 18) stakeholders) Age: Phase 1, 6‐13 years	15 min per school day for 12 weeks, Phase 1 Phase 2, January‐March 2017	**Case series with post‐test outcomes** **IV** 2‐phase multi‐method process evaluation. Phase 1—75 student intervention. Phase 2—focus groups of 18 stakeholders *Measures*: structured observations, Children's OMNI perceived exertion scale at 4 time points, System for observing fitness instruction time (SOFIT). MVPA levels	During 12‐week teacher delivered implementation 93.6% of time. Approximately 95% students participated, completed recommended 15 minutes and engaged in MVPA. 3 key emergent themes: embedding intervention, right physical environment and supportive relationships and climate	Yes **(Involve)**	No
45 UK	Switch Off—Get Active!	Harrison, Burns^96^	**T:** Not reported **HPF**: Not reported	↑ PA ↓ Screen time	School: (n = 9, IG and CG) Student: (n = 312, IG 182 and CG 130 Age: 9‐11 years	10 × 30 minute lessons 16 weeks, February‐June 2003	**A comparative study with concurrent controls—case‐control study** **III‐2** **T1:** Baseline **T2:** Post *Measures*: 1‐day previous day. Physical Activity Recall (PDPAR) × 3, physical activity self‐efficacy, BMI (kg/m^2^), aerobic fitness 20 m shuttle test, diaries with activity point system	↑ Significant PA +0.84 30‐minute blocks/d and self‐efficacy for IG NS difference between pre‐post screen time, BMI and aerobic fitness for IG and CG individuals At school level significant ↑ PA in all IG and CG. Significant ↓ Screen time in 4/5 IG and 2/5 CG	No	No
46 UK	HPS in Galway	John‐Akinola and Gabhainn^97^	**T**: Theory driven—socio‐ecological model **HPF:** HPS, WHO Ottawa Health Charter	↑ Health ↑ Well‐being	Schools: (n = 9, 4 DEIS and 5 non‐DEIS) Students: (n = 231, DEIS 139 and non‐DEIS 84) Age: 9‐13 years, G4‐6	Not reported	**A comparative study with concurrent controls—case‐control study** **III‐2** *Measures*: School socio‐ecological environment, Health behavior in school‐aged children (HBSC) 4 items	No significant differences between HPS and non‐HPS schools for health and wellbeing (data not reported)	No	No
47 UK	National Healthy Schools Program (NHSP)	Keyte, Harris^98^	T: Not reported **HPF**: National Healthy Schools Program (NHSP)	↑ F&V intake	Schools: (n = 10, 7 NHSP and 3 non‐NHSP) Students: (n = 511, NHSP 410 and non‐NHSP 101) Age: 7‐9 years	Not reported	**A comparative study with concurrent controls** **III‐2** *Measures*: Day in the Life Questionnaire (DILQ)—24‐hour dietary recall Varying levels of engaged NHSP status	F&V intake for Engaged NHSP was significantly more than non‐engaged students (2 cf. 1 portion) Gender was a significant predictor of F&V consumption. Girls 1.68 times more likely to consume	No	No
48 UK	Active for Life Year 5 (AFLY5)	Kipping, Howe^99^ Anderson, Howe^100^	**T:** Not theory driven—social cognitive theory (SCT) **HPF:** Not reported	↑ PA ↑ Sedentary behavior ↑ F&V intake	Schools: 60, IG and CG Students: (n = 2221, IG 1064 and CG 1157) Age: 8‐10 years, G4‐5	16 lessons over 6‐7 months	**Cluster RCT** **II** Process evaluation **T1**: Baseline year 4 or early year 5 **T2:** Post‐intervention end of year 5 *Measures*: Accelerometers for PA and sedentary time (5 days, 3 weekdays, 2 weekends), Anthropometric BMI (kg/m^2^), waist size, Day in the Life Questionnaire (DILQ)—24‐hour dietary recall	No significant effect found on ↑PA, ↑sedentary behavior or ↑F&V intake Long‐term follow‐up NS of 3 outcomes between groups IG and CG School‐based interventions alone unlikely to have a major public health impact on children's diet PA	No	No
49 UK	Healthy Lifestyles Program (HeLP)	Lloyd, Wyatt^101^ Lloyd, Creanor^102^	**T:** Theory driven—behavior change theory, information motivation and behavioral skills models **HPF:** HPS	Obesity prevention program ↓ Sweetened fizzy drinks ↑ Healthy snacks ↓ Screen time	Schools: (n = 4, IG 2 and CG 2) Students: (n = 202) Age: 9‐10 years	3 Terms: Spring and Summer term, year 5 and Autumn term, year 6	**Cluster RCT** **II** **T1**: Baseline **T2:** Start of school year, Autumn **T3:** 12 months **T4**: 24 months *Measures*: Anthropometric BMI (kg/m^2^), waist size, Actigraph for PA, Food Intake Questionnaire—24‐hour recall × 2, Children's TV Viewing Habits Questionnaire	Anthropometric measures ↓ in IG than CG at T3 and T4, except body fat %. T3 6% increase in overweight and obese in CG, while IG remained at T1 At T3 IG had ↓ sweetened fizzy drinks, ↑ healthy snacks and ↓ screen time cf. CG Follow‐up: 24 months NS effect on ↓ obesity	Yes (children, parents, school staff) **(Collaborate)**	No
50 UK	Strathclyde Evaluation of Children's Active Travel (SE—CAT) Traveling Green	McMinn, Rowe^103^ McMinn, Rowe^104^	**T:** Not theory driven—theory of planned behavior (TPB) **HPF:** not reported	↑ PA	Schools: (n = 5 schools, IG 2 and CG 4) Students: (n = 166, IG 79 and CG 87) Age: G5	6 weeks active	**A comparative study with concurrent controls** **III‐2** **T1:** Baseline **T2:** Post **T3**: Follow‐up 5 months **T4:** Follow‐up 12 months *Measures*: Actigraph for PA Child and parent Questionnaire for travel, Travel diary	Pilot study of feasibility and measures—data analysis not completed in this study Follow‐up: little effect on school walking Significant ↑ steps and MVPA between IG and CG.	No	No
51 UK	Spring fever	Newby and Mathieu‐Chartier^105^	**T**: Not reported **HRF:** Not reported	SH Assessing: reach, program fidelity, dose, recruitment, and context	Schools: (n = 1) Students: (n = 302), of which 24 provided feedback Age: 4‐11 years	1 week to all school years	**Case Series with post‐test outcomes** **IV** Process evaluation Feedback from Teachers (forms and focus group), parents (forms, daily diary, and interviews), and students (small group interviews)	Reach—high Fidelity—high Dose—varied (high G1‐G4, low G5‐G6) satisfaction reported for teachers, parents but not students Context—sensitivity as delivery dependent upon teacher and student	Yes **(Consult)**	No
52 UK	Kids, Adults Together (KAT) Program	Rothwell and Segrott^106^ Segrott, Rothwell^107^	**T:** Theory driven—social development model **HPF:** not reported	↓ Alcohol intake	Schools: (n = 2) Students: (n = 54) Age: 9‐11 years	1 week with afterhours KAT event	**Case Series with pre‐/post‐test outcomes** **IV** Pilot evaluation **T1:** Baseline **T2:** Post *Measures*: Classroom observation × 2, KAT event observation × 1, staff interviews × 1, focus groups × 2, parent interviews × 2, parent questionnaires × 1	High level of acceptability and involvement of children and parents, 50% of parent participated. Perceived impact on: ↑ pro‐social communication within families, ↑ knowledge and awareness, changes in parental drinking behaviors. Key criteria for effectiveness trial not met	Yes **(Collaborate)**	Yes
53 UK	The CLASS PAL (Physical Activity Learning) Program	Routen, Biddle^108^	**T:** Theory driven—COM‐B model of behavior Behavior change techniques **HPF:** not reported	Assessing intervention on ↑ PA on: implementation (fidelity, dose, and quality) at individual ad school level	Schools: (n = 6) Students: not yet recruited in study Age: G5	Not reported	**A comparative study without concurrent controls—interrupted time series without a parallel control group** **III‐3** **TI**: Baseline, October 2016 **T2**: December 2017‐March 2017 **T3:** April 2017‐May 2017 **T4:** June 2017‐July 2017 **T5**: September 2017‐October 2017 *Measures*: Teacher and school characteristics, Pupil questionnaire and anthropometric data Classroom observations T1 and T4, Pupil focus groups and teacher interviews T2, T4, and T5 Actigraph for PA, International Physical Activity Questionnaire School Physical activity, Promotion Competence Questionnaire, Adolescent Sedentary Activity Questionnaire, Engagement versus Disaffection with Learning Scale, d2 test	Evaluation design protocol	Yes—school stakeholders in development phase **(Involve)**	No
54 UK	Active Program Promoting Lifestyle Education in School (APPLES)	Sahota, Rudolf^109^	**T:** Not reported **HPF**: HPS	Obesity prevention program	Schools: (n = 10, IG 5 and CG) CG received the next year) Students: (n = 634) Age: 7‐11 years	12 months	**Group RCT** **II** **T1**: Baseline **T2:** Post 12 months *Measures*: Questionnaires (students, staff and parents), training evaluation, School action plan implementation	Evaluation design protocol CG included in baseline data with IG—no differentiation between group differences 76/85 School action points implemented Positive ↑ changes in 5 foods offered	Yes—schools, teachers, parents, caterers, and pupils **(Involve)**	No
55 UK	The Primary Drama Drug Project	Starkey and Orme^110^	**T:** Not reported **HPF**: Not reported	↓ A&T, and illegal drug uptake	Schools: (n = 6) Students: (n = 297 pre and 253 post) Age: 10‐11 years	1 day, followed by 4 workshops and student performance	**Case Series with pre/post‐test outcomes** **IV** Process and impact evaluation **T1**: pre **T2:** post 1‐month *Measures*: Draw and Write exercise Alternatives and Consequences test	Intervention made a positive contribution to knowledge, education and parental involvement. ↑ in student problem solving skills post	No	No

* Refer to Table 2 for selected Social Impact evidence statements.

Abbreviations: T, theory; HPF = health promoting framework; PA, physical activity; MVPA, moderate vigorous physical activity; N, nutrition; HE, healthy eating; HL, healthy living; ENE, enhanced nutritional education; T&A, tobacco and alcohol; F&V, fruit and vegetable; LNDF, low‐nutrient density foods; AOP + T = Aussie Optimizing Program + Teaching; AOP + T&C, Aussie Optimizing Program + Teaching and Coaching; SLS, social life skills; OTS, optimistic thinking skills; OCHP, Ottawa Charter for Health Promotion (1986); CSH, Comprehensive School Health; CGSHE, Canadian Guidelines for Sexual Health Education; NIDDM, non‐insulin‐dependent diabetes mellitus.

### Social Impact Versus Outcomes

Social impact was rarely measured in the interventions. Only 16 studies (29%) indicated they had considered or attempted to measure social impact. Social impact was not clearly understood and described in interventions, and often anecdotally and qualitatively measured (see Table 2). Outcomes of the interventions (the results or effects of a program and the changes that occur in attitudes, values, behaviors, or conditions of interventions) were measured rather than the social impacts (the economic, social, and environmental consequences, positive or negative, regardless of the purpose or perceived or real benefits of the activity)^20^ or theory used. Two interventions (4%) alluded to social impact being a justification for the intervention. Eight interventions (15%) measured or attempted to measure aspects of social impact, although had not comprehensively measured the impact of the interventions. Of these 8, 4 mentioned that there was a positive impact on families or communities, individual's knowledge or benefit beyond the program. However, it should be noted that measurement was not methodical (assessing against a framework, theory, or program logic) nor was it systematic (assessing all potential impacts—positive, negative intended or unintended) nor comprehensive (examining impacts in multiple domains such as individual, societal, economic, and policy levels). There were 8 studies (15%) which alluded to the broader social, environmental, or economic impacts of the intervention beyond the reportable outcomes of the intervention but made no mention of measuring this social impact. This review found only (14%) of interventions were implemented over 2 years (14%), with a wide variance in dosage, intensity, and delivery. Overall, interventions were not assessed systematically against a framework, theory, or program logic, nor were changes in the broader societal, economic, and policy determinants effectively considered.

**Table 2:** Social Impact Location and Evidence

Attempted measurement of social impact				
No	Study	Study	Social Impact mentioned	Location and evidence
1 AU	Play Zone in Primary Schools	Austin, Caperchione^37^	Social	“Furthermore, 86% (6/7) of the implementing schools reported noticeable changes in children's behaviors other than PA, as a result of the intervention. These behaviors included reductions in fighting, reductions in boredom and disruptive behaviour during school breaks, and increased incidents of cooperation, negotiation, and sharing” (p. 937) “They're incidentally learning all the time, and the other thing is that you're taking kids away from being in the situation where they're going to have antisocial behavior; they're having success and they're happy” (p. 937)
3 AU	Go for your life!	de Silva‐Sanigorski, Prosser^39^ Honisett, Woolcock^40^	Social Environment	Community and organization: “Improved policy and practices; Improved community links and partnerships; Health promoting environments; Improved knowledge, skills, beliefs, perceptions” Family: “Increased physical activity‐related behaviours; Increased healthy eating; Increased knowledge, skills, beliefs, perceptions” Child: “Increased healthy weight; Decreased obesity; Increased quality of life” (p. 3) (Figure 1)
4 AU	Kids Matter Primary	Dix, Slee^41^ Graetz, Littlefield^42^	Social	“In brief, the questionnaires sought information on areas of school engagement and implementation of the initiative, impact on the school in general, impact on teachers and families, and impact on student social‐emotional competence and on their mental health” (p. 47) “…to collaborate on KidsMatter with the aims of improving the mental health and well‐being of students, reducing mental health problems…” (Graetz et al 2008, p. 15)
7 AU	Stephanie Alexander Kitchen Garden Program (SAKGNP)	Eckermann, Dawber^47^ Gibbs, Staiger^48^	Social Environment Economic	“Assessing multiplier impacts from investment on related community activity over time are suggested as key alongside evidence of program health effects on targeted groups of individuals in gauging community network engagement and ownership, dynamic impacts, and program long term success and return on investment … impact on total community activity up to two years was 5.07 ($226,737/$44,758); 1.60 attributable to school, and 2.47 to wider community, activity” (p. 103)
23 CA	Action Schools! BE—healthy eating (AS! BC—HE)	Day, Strange^68^ Naylor, Macdonald^69^	Social Environment Economic	Figure 2 “↑long term health outcomes…, ↑child and youth achievements, ↓chronic disease, ↓health cost, healthier kids” (p. 5) “Impact at the systems level is measured by changes in public policies or organizational practices including legislation, funding, procedures, regulations, and incentives” (p. 5) “…enhances the impact and sustainability of health promotion initiatives” (p. 6)
25 CA	Kahnawake Schools Diabetes Prevention Project	Macaulay, Paradis^71^ Adams, Receveur^72^	Social Environment	“The Precede‐Proceed model identifies predisposing, reinforcing, and enabling factors, as well as environmental and organizational factors, that impact on health behaviors. For KSDPP predisposing factors are children's knowledge and skills, reinforcing factors are the support of teachers and family, and enabling factors are the availability of healthy foods and opportunities for physical activity” (p. 8) “Community‐based interventions improved children's lifestyles” (Adams et al 2005, p. 404)
33 UK	WAVES	Adab, Barrett^7^ Clarke, Griffin^81^	Economic Social	“The aim was to estimate the cost‐effectiveness of an obesity prevention intervention program in primary school‐aged children” (p. 99) “(Teacher) Question 6: overall, what impact (if any) do you think the WAVES study intervention program had on your year 2 children? (Parent) ‘Question 5: what did you think of the cooking workshops? Do you think the workshops had any impact on your family?’” (p. 35) “…in terms of the perceived impact. Families from higher socioeconomic areas considered that they gained little additional knowledge and already practised healthy behaviours, whereas positive lifestyle changes were more likely to be reported by families from more disadvantaged communities” (p. 125) “…although school is an important setting for influencing children's health behavior, wider impacts from the family and community, including socioeconomic circumstances, must also be considered” (p. 124)
52 UK	Kids, Adults Together (KAT) Program	Rothwell and Segrott^106^ Segrott, Rothwell^107^	Social	“…perceived impacts of the programme were increased pro‐social communication within families (including discussions about harmful parental alcohol consumption), heightened knowledge and awareness of the effects of alcohol consumption and key legal and health issues, and changes in parental drinking behaviours … through its impact on knowledge and communication processes within families” (p. 1) “A range of health and social impacts of alcohol misuse by young people has been documented, including disorderly and violent behaviour, risky sexual behaviour [1], accidental injury, poor school attendance and achievement… The global costs of alcohol misuse related to such impacts are high” (p. 2)
**Mentions social impact**				
2 AU	Live Life Well@school	Bravo, Foley^38^	Social Economic	“Childhood obesity is a global public health issue, which has profound health, economic and social impacts” (p. 2)
19 AU	Aussie Optimized Program (AOP)	Roberts, Williams^62^		“…assessing the impact of AOP on health risk behaviours” (p. 80)
22 CA	Healthy Buddies	Campbell, Barnum^66^ Ronsley, Lee^67^	Social	“Healthy Buddies_ improves knowledge not only in Gr. 4‐7 students but also in their younger, K‐Gr. 3 buddies, which may have a positive impact on behaviours, attitudes and habits” (p. 186)
26 CA	Passport: Skills for Life (PSL)	Mishara and Dufour^73^	Social Environment	“…were asked what was their perception of the impact of the program on children, the class and school environments” (p. 8)
34 UK	Marathon Kids	Chalkley, Routen^82^	Social	“It was apparent that pupils valued the shared experience and sense of community MK provided” (p. 54) “A pervasive finding from the data was the sense of social connectedness across the whole school, which was evident during, and subsequent to, the schools' participation in MK” “…MK resources provided by KRF, these were used … may have negatively impacted on pupils' enjoyment of the program” (p. 56)
37 UK	Project Tomato	Evans, Ransley^88^	n/a	“There appears to be no long‐term impact of this scheme on fruit and vegetable intake of children” (p. 1073)
42 UK	The Lifeskills Program	Gabhainn and Kelleher^93^	Social	“…an important impact of Lifeskills on drinking behaviour among young people and suggest that the programme makes a positive contribution…” (p. 599)
49 UK	Healthy Lifestyles Program (HeLP)	Lloyd, Wyatt^101^ Lloyd, Creanor^102^		“We believed that the cumulative effect of making small sustainable changes in multiple behaviours related to the energy balance had the potential to significantly impact on weight status” (p. 10)

### Theory and Health Promoting Frameworks

Many interventions were not informed by theory (n = 27, 49%), with a further (n = 7, 13%) found to be *not theory driven* and were instead theory informed interventions (mentioning theory but failing to apply a theoretical framework in the study components or measures).^111^ Interventions were also found not to report any health promotion framework (n = 34, 62%). Theories and frameworks are needed to inform and describe what we do, and guide effective implementation of interventions. Theoretically informed and measured interventions show how the targeted behavior(s) were: (1) informed by theory, (2) had theory applied, (3) theory tested, or (4) built upon theory.^25^, ^111^ Some interventions mentioned more than 1 theory or health promotion framework. Of those which referred to theory, the majority were social based theories (theories which examine the social influences on people, environments, and behaviors)^112^ (n = 17, 31%), the most common being the socio‐ecological model (SEM) (the wider multilevel influences on individual behaviors such as the culture and environmental settings, policies, and engagement with the wider community)^39^ (n = 7, 13%), and social cognitive theory (SCT) (individual's knowledge acquisition is associated and influence by the observation of others during social interactions and experiences and recognizes personal and socio‐structural determinants of health)^113^, ^114^ (n = 3, 5%). Behavioral‐based theories (such as The Behavior Change Wheel and COM‐B Framework)^115^ (n = 6, 11%) and psychological theories (such as Self‐Determination Theory and Competence Motivation Theory) (n = 5, 9%) were the next most common.

### Stakeholder Engagement

Over half of the 55 intervention studies identified some level of stakeholder engagement. No studies engaged with stakeholders at the lowest level (*Inform*); 8 studies (14%) were rated at the *Consult* level; 12 studies (22%) were rated at the *Involve* level; 9 (16%) studies were rated at the *Collaborate* level, 1 study (2%) was rated at the *Empower* level; and 26 interventions (46%) did not report stakeholder engagement.

## DISCUSSION

The aim of this review was to understand how social impact was considered and measured in children's primary school health promotion interventions in 4 comparable countries. To achieve this, both the behavioral focus of children's health promotion interventions, and the application of theory and/or health promotion within these interventions was examined, along with examination of how social impact was considered or measured. Although behavioral focus was strong, application of theoretical and health promotion frameworks occurred in less than half of the studies, and consideration and measurement of social impact was limited. If studies do not have strong behavioral effects, and create the predicted behavioral change, it is unlikely interventions will create social impacts, particularly for interventions which had weak or no effects.

Social impact was not always considered nor clearly measured within the primary school health promotion programs in this review. Of the studies that inferred or attempted to measure some form of impact, it was more likely to be the social benefit of the intervention. This necessitates distinction between an intervention's positive impact (a positive effect or improvement on a behavior or measure)^116^ and the broader social benefit (how society is better off when there is a behavioral change creates benefits or decreases harm)^117^ before social impact can be measured. The social impact resulting from interventions was often unclear for several reasons including: (1) the social impact that the intervention aimed to produce was not considered in the intervention design, and if considered it was as an “impact” on behaviors which affect health or social well‐being or knowledge acquisition; (2) a lack of clarity in how to incorporate and measure social impact; and (3) social impact if explored, was often through a qualitative means whereby participants were asked about the “impact of the program” with subjective open‐ended questions examining what changed as the result of the intervention.

Theories of change explain how activities are understood to produce a series of results that contribute to achieving intended impacts providing an explanation of how and why a program works.^118^, ^119^ This guides intervention development and delivery and ensures that the critical components needed to achieve change are included. The low level of theory use and rare application of health promotion frameworks within these health promotion interventions was concerning. Health promotion aims to influence the broader benefits at the social, environmental, policy, or economic levels.^120^ Without theoretical guidance, important components can be omitted, and interventions may then fail to achieve the desired outcomes that create broader impact. Importantly, without theoretical explanation, it is not clear why interventions have succeeded or failed which prevents replication or duplication in other settings.

Health promotion in primary school settings often targets complex behavior, whether it is addressing a singular behavior such a not starting to smoke, or addressing multiple behaviors within a domain, such as healthy eating (increasing fruit and vegetable consumption, providing healthy eating skills) or physical activity (increasing steps taken per day, decreasing screen time). However, in this review, complex interventions conducted by Kipping, Howe,^121^ and Ofosu, Ekwaru,^76^ which targeted multiple behaviors, found no change in individual health determinants. Complex interventions need to measure social and economic health determinants, such as health equity,^122^ access to healthy foods or safe exercise environments^123^, ^124^ to be able to capture social impact.

The interventions which most clearly applied and measured social impact in children's primary school health promotion were more likely to have used a theoretical lens (social or behavioral based) and generally were informed by a health promotion framework. These lenses should encourage consideration of the broader effects of the intervention. Social impact is rarely measured as these broader effects are not being measured, even when SEM theories or HPS frameworks are reported. Whether it is a program logic such as Naylor, Macdonald^69^ or an alternative logic model,^125^ interventions need to provide a clear explanation of what the intended goal of the program is, outline the predicted outputs or outcomes and explain why a program is expected to work. Effective evaluation requires health promotion interventions to have stronger use of theory or health promotion frameworks to understand and map where and how change is occurring, or not occurring, rather than solely whether the input has created the desired outputs. This underpins effective intervention delivery and measurement, with an identification of the short‐ or long‐term impacts and consideration of the intended and unintended consequences, both positive and negative, of programs socially, economically, and environmentally.^45^, ^126^

Evaluation of research and programs creates a map of how the research/program has worked in practice and provides key information about effective and ineffective practices and process, allocation of resources and sustainability.^127^ Brief interventions neither capture sustained behavior change^28^ nor target the structural issues which reinforce or drive complex wider issues such as obesity and mental health. If health promotion is to deliver lasting changes, evaluation of interventions requires more than measuring inputs, outputs, and outcomes of individual health determinants. Incorporation of broader social, community, and ecological measures in health promotion evaluation is required to measure and demonstrate what changed, and if it has changed differently for different individuals or groups, as competition for resources, funding, and time allocation within schools are rapidly increasing.^128^ Polonsky, Landreth Grau,^129^ and Nicholls^130^ highlight the need for more effective ways to utilize resources and address social issues to improve social outcomes. Health promotion needs to strive toward being more accountable in the way that delivery and demonstrated impact can be accurately estimated and clearly communicated.^131^

## LIMITATIONS AND FUTURE RESEARCH DIRECTIONS

The findings of this review should be considered in light of its strengths and limitations. This review included a large number of studies across multiple countries, providing a strong platform to assess social impact within health promotion in primary schools. The first important limitation is the generalizability of the findings as countries outside of the 4 Commonwealth countries, multiple countries, and systematic reviews were excluded from the analysis which may have yielded additional insights. A related limitation is the Anglo‐centric focus, with non‐English studies excluded, findings may not be representative of other cultures. Future research could replicate this examination in a broader group of countries. In addition, the search parameters used for this review, may have created a bias on which interventions were included and excluded from the analysis, meaning the studies are not an exhaustive list of health promotion interventions, programs, or initiatives conducted in primary school settings in Australia, Canada, New Zealand, or the United Kingdom. This review established a low level of social impact measurement within a large pool of studies, indicating a strong need for social impact measurement in school health promotion. To advance effective health promotion, future research needs to address the barriers to implementing interventions which measure social impact. If we are to understand the value of measuring what has changed beyond the individual, clear mapping of the behavioral focus from input to outcome, stakeholder engagement, and the measurement against theoretical constructs, needs to occur before a social impact chain can be established. Currently, the paucity of social impact research within this context of health promotion interventions limits understanding of the broader social, economic, and health benefits of primary school health promotion, and social impact remains poorly defined, misunderstood, or not measured. A greater level of research in this area will contribute to better understanding and measurement.

## CONCLUSION

Interventions should evidence how they create benefits by measuring the social impact, short or long term, whether societal, environmental, or economic benefits or a combination of these 3 benefits.^132^, ^133^ Social impact measurement allows the return on investment for programs to be clearly communicated, supporting well‐informed funding decisions to be made. There is a need for social impact to be incorporated and evaluated in primary school health promotion interventions to provide evidence of the benefits these interventions create and to demonstrate “value for money.”^134^ This review indicated social impact measurement is poorly understood and measured. Greater understanding is needed, and clear mapping of theory onto programs is required to explain why change occurs, and how this change leads to social impact.^135^, ^136^ Only then can social impact measurement be embedded as a standard practice within health promotion interventions, programs and initiatives.

## Implications for School Health

Health promotion efforts in primary schools need to be evaluated to measure whether they are effective in promoting behavioral changes in children, and whether they establish longer term safe and healthy life choices. This review sought to examine how social impact was measured within primary school health promotion interventions and found significant gaps in how social impact was understood, and how it was measured. In addition, theoretical and health promotion frameworks were often poorly implemented or considered in the evaluation of the intervention.

This review suggests to achieve more effective health promotion in primary schools, design, implementation, and evaluation needs to consider the following:

- Examination of the social impact of projects, programs or initiatives offers a means to understand the value of improved health behaviors and outcomes for school community stakeholders such as students, schools, and parental school communities.^4^
- Evaluations should be grounded in theoretical determinants^111^ to understand whether determinants of change have altered as a result of intervention, and to measure the individual and the broader societal impact resulting from children's health promotion. From a social impact estimation standpoint, theory delivers the understanding needed for clear attributions to be made. In the absence of causal links explaining the size of the effect, attributions are little more than a guessing game.
- Evaluation needs to involve greater stakeholder engagement, to establish and measure the social impacts of an intervention, to understand if health promotion is effective within schools. Interventions need to target what matters to key stakeholders and encourage active participation if effective behavioral change is to be achieved.
- Where possible, interventions need to plan for longer durations or frequent dosage. Stakeholder engagement and longer duration interventions are costly and resource intensive, and funding at this level not always available. However, to deliver broader social impact and evidence health promotion actions provide value for money, consideration of intervention length, dosage and the level of stakeholder engagement are important.

Moving forward, it is important that interventions in primary schools consider when measuring outcomes or social impact: What *impact should they see*; what *impact has occurred* and the mechanisms; what *types of impact* have occurred; *who has been affected or impacted*, and to how to *evidence impact* for impact measurement models.^22^, ^24^ This ensures funding is directed to programs that deliver lasting change that benefits individuals and achieves improved health outcomes and cost savings for societies that fund health promotion efforts.

### Human Subjects Approval Statement

Preparation of this article did not involve original research or data collection with human subjects.

### Conflict of Interest

The authors declare no conflict of interest.

Open access funding enabled and organized by Projekt DEAL.

## Supporting information


**Appendix** **S1**: Supporting Information
