# Supplementary material for: Evaluation of Social Impact Within Primary School Health Promotion: A Systematic Review
Source: J Sch Health. 2022 Apr 1;92(8):739–64. doi: 10.1111/josh.13160 (PMC9544285; doi:10.1111/josh.13160)
Supplement: Supplementary file 1 — Appendix S1: Supporting Information [file JOSH-92-739-s001.doc]

| **Reviewer** | **Reviewer Comment** | **Author’s response** | **Corrections** | **Location of correction** |
| --- | --- | --- | --- | --- |
|  | **IN GENERAL** |  |  | **Track changes p.? (simple mark up p?)** |
| 1.1 | The paper could use a more focused approach to the research question – if/how social impact is measured; OR is the focus broader than what is alluded to in the aim?  Eg. To look at what types of studies were more likely to include measuring social impacts? At times the paper seems to wander and not be clear on what the intent is or the connections between factors. | Thank you for the opportunity to clarify the aim of the paper. | We have addressed this throughout the paper. E.g.1.2, 1.6, 1.8, 1.9 (7), 1.12, 1.13, 1.14 1.15, 1.16, 1.17, 1.18, 1.21,1.22, 1.23. 1.24 |  |
| 1.3 | Not sure why these 4 countries were selected; and, if most of the included studies did not measure social impact, maybe looking at what types of studies did include social impact across a broader number of countries would be more informative? | Addressed in methods and limitations  We have acknowledged the limitations of focusing on these four countries | Refer to actions undertaken in 2.3  … limitation is the generalisability of the findings as countries outside of the four Commonwealth countries, multiple countries and systematic reviews were excluded from the analysis which may have yielded additional insights. | p.11-12 (p.11) |
| 1.4 | Another issue is that of the few studies that measured/attempted to measure social impacts, there are really no ‘results’ presented for what those were. | Thank you for this insight. The authors have addressed this by clarifying that this is the very issue of social impact within health promotion – it is not well understood nor well measured | see actions undertaken for reviewer comment 1.8, 1.9, 1.11, 1.12 |  |
|  | **TITLE** |  |  |  |
| 1.2 | Title: I am not sure the title accurately reflects the paper focus; the review looks at whether school based health program evaluations included social impact as an outcome; and it is not really across 4 countries as only 2 were from NZ, and this (differences, similarities) is not addressed at all in the paper. | Thank you for this feedback.  We have changed the title to better match the paper focus.  We have justified the inclusion of four countries in the methods section. | We have changed the title to:  Evaluation of social impact within primary school health promotion: A systematic review  Primary school health promotion interventions were sought from four Commonwealth countries deemed to have comparable health systems and similar approaches to public health prevention and health promotion in schools. | p.1 (p.1)  p.4 (p.4) |
|  | **ABSTRACT** |  |  |  |
| 2.1 | In the Abstract, elaborate on what is meant by "social based theories." | Thank you for the opportunity to add a clearer definition. | We have added a definition in the abstract and in the text for social based theories (theories which examine the social influences on people, environments, and behaviours) and added an appropriate citation | p.1 (p.1)  p.8 (p.7-9) |
|  | **INTRODUCTION** |  |  |  |
| 1.6 | Pg2 – The authors acknowledge the shortcomings already described in the literature about school-based health promotion evaluations, and seem to conclude the same in the paper/current study | This paragraph, and the one below, are used state what the focus of previous reviews has been (on outcomes), which is different to that of this study (on social impact). We have added text here to show these reviews are valuable (not shortcomings). | Whilst these reviews are important for establishing how programs lead to direct, measurable outcomes, they do not increase understanding of how programs may lead to social impact. | p.4 (p.4) |
| 1.7 | Pg3 – The authors acknowledge that evaluations of school-based health programs focus on behavioral outcomes and that review of social impact are lacking, once again similar to their own findings | See above.  The revised text strengthens the point that reviews are lacking, and articulates that need for this review. | See above |  |
| 2.2 | On page 3, define/elaborate on "anthropmetric change." | Thank you for the suggestion. We have added a definition with a citation. | anthropometric measurement (standard body measurements such as such as weight, height, skinfolds and waist circumference).^1, 2^ | p.3 (p.3) |
| 1.8 | Pg4 – ‘The purpose of the review is threefold: firstly, to examine the reporting of theoretical or health promotion frameworks in the health promotion interventions (WHY? How is this connected to social impacts); secondly, to understand how social impact is considered and measured in health promotion; and finally, to highlight the key learnings for social impact measurement in health promotion for primary school children for future health’ – this does not seem to be focus of paper | We have revised the purpose to better match the aim and outcomes of this paper | The purpose of the review is twofold: firstly, to understand how social impact is considered and measured in health promotion; and secondly, to highlight the key learnings for social impact measurement in health promotion for primary school children to guide future health promotion interventions. | p.4 (p.4) |
|  | **METHODS** |  |  |  |
| 2.3 | Justify why the four "commonwealth countries" were included in the study, provide background on how they are linked. | Thank you for the suggestion. We have added a justification for why we selected those four countries in the methods. | Primary school health promotion interventions were sought from four Commonwealth countries deemed to have comparable health systems and similar approaches to public health prevention and health promotion in schools. | p.4 (p.4) |
| 2.4 | Please add any reliability data for interobserver agreement reviewed on page 5. | Thank you for your feedback. The number of issues resolved was added. | when disagreements (*n* = 4 issues) were encountered | p.5 (p.5) |
|  | **RESULTS** |  |  |  |
| 1.9 | Recorded the following for the 55 selected interventions: … 2) type of theory and/or health promotion framework reported, 3) behavioural focus/intervention approach of the health promotion intervention, 4) sample set used in the evaluation, 5) duration of the intervention, 6) type of evaluation study design and methods used for intervention, 7) the reported outcome effects/results of the intervention, 8) the level of stakeholder engagement in the intervention and, 9) and presence and nature of any social impact measurement. – But in results only discuss 7) evidence/3) behavior focus; 2) theory/framework; 8) stakeholder engagement; and 9) social impact. | Thank you for the comment. Please find that we have addressed each missing points. | 4) sample set used in the evaluation:  Sample size and duration also demonstrated this same variation. Sample sizes ranged for students (from ‘not reported’ to 4808) and schools (1 to 193) making meaningful comparison equally challenging.  5) duration of the intervention:  This review found only (14%) of interventions were implemented over 2 years (14%), with a wide variance in dosage, intensity and delivery.  6) type of evaluation study design and methods used for intervention:  Most of the evaluation study designs were RCTs (cluster or groups) (36%), cases studies (30%), or comparative studies (with concurrent controls) (21%) and without concurrent controls (7%). Regardless of study design, most conducted pre-post/post-test. Interventions were a mix of process evaluations (13%), process and outcome evaluations (5 %), impact evaluations (5%) , a mixture of process, outcome or impact evaluations (4 %), RE-AIM evaluations (2%) or a realist evaluation (2 %).  7) outcome effects/results of the intervention:  However, the variation in outcomes measured makes it difficult to draw any conclusions on whether these interventions resulted in social impact. | p.6 (p.6)  p.7 (p.7)  p.6 (p.6)  p.6 (p.6) |
| 1.10 | P5 – ‘Evidence levels, in accordance to the NHMRC evidence hierarchy, found strong evidence at level II (N = 20, 36%), moderate evidence at level III-2 (n =12, 21%) and level III-3 (n = 7, 13%) and weak evidence at level IV (30%, n = 17).’ Evidence of changing the targeted behavior? | Thank you for your comment. We have revised the text in the methods to better represent how the NHMRC assessment is intended to be used.  We have then revised the text in the results to give a clearer interpretation of the findings of the assessment process. | The National Health and Medical Research Council’s (NHMRC)’s quality assessment framework was used to grade the study evaluation design used in each intervention, from I (highest) to IV (lowest) to assess the level of evidence each evaluation can contribute to the evidence base.  The NHMRC evidence rating process rated 20 studies at level II (36%), 12 studies at level III-2 (21%) 7 studies at level III-3 (n = 7, 13%) and 17 studies at level IV (30%). This represents a body of evidence of sufficient size and quality to be able to guide practice. | p. 5 (p.5)  p. 6 (p.6) |
| 1.11 | P6 – ‘The interventions were not always informed by theory (n = 34, 62%), with 27 of these not reporting the theory used, and a further 7 were not theory driven’ – Not clear on the difference | We have revised the text to clarify which studies were not informed by theory, which were theory informed, and which were theory driven. | Many interventions were not informed by theory (n = 27, 49%), with a further (n = 7, 13 %) found to be ‘*not theory driven*’ and were instead theory informed interventions (mentioning theory but failing to apply a theoretical framework in the study components or measures). | p.7-8 (p.7)  Table 1 |
| 1.12 | Should results include what types of studies were more or less likely to include social impacts? | Thank you for the comment. | Overall, interventions were not assessed systematically against a framework, theory or program logic, nor were changes in the broader societal, economic and policy determinants effectively considered.  Social impact is rarely measured as these broader effects are not being measured, even when SEM theories or HPS frameworks are reported. | p. 7 (p.7)  p.11 (p.10) |
| 2.5 | On page 6, enhance description of and provide references for the theories described. | Thank you for your feedback. We have added enhanced descriptions with citations for each of the described theories. | the Socio-Ecological Model (SEM) (the wider multi-level influences on individual behaviours such as the culture and environmental settings, policies, and engagement with the wider community),  Social Cognitive Theory (SCT) (individual's knowledge acquisition is associated and influence by the observation of others  during social interactions and experiences and recognises personal and sociostructural determinants of health) ^3, 4^ psychological theories (such as Self Determination Theory and Competence Motivation Theory) Behavioural based theories (such as The Behaviour Change Wheel and COM-B Framework)^5^ | p.8 (p.8) |
|  | **DISCUSSION** |  |  |  |
| 1.5 | Stated Aim: ‘The aim of this review was to understand how social impact was considered and measured in children’s primary school health promotion interventions in four comparable countries.’ – Bottom line only 8 of 55 measured/attempted to measure. But authors also discuss behavioral outcomes, theory, framework, stakeholder engagement – how does this fit into the Aim? The results section does not identify what types of studies were more or less likely to include social impacts. | The authors thank you for your comments and we have addressed in two parts. | See actions undertaken for reviewer comment: 1.2, 1.6, 1.8, 1.11, 1.15, 1.17, 1.20, 1.22, and 1.23. |  |
| 1.13 | P7 – ‘Of the studies that inferred or attempted to measure some form of impact, it was more likely to be the social benefit of the intervention.’ – Explain difference between social benefit and impact. | Thank you for the opportunity to further explain the difference. | measured (see Table 2).  This necessitates distinction between an intervention’s positive impact (a positive effect or improvement on a behaviour or measure)^6^ and the broader social benefit (how society is better off when there is a behavioural change creates benefits or decreases harm)^7^ before social impact can be measured. | p.9 (p.9) |
| 1.14 | Pg8 – Paragraph on theory – do not see an explanation of how this is related to social impacts | Thank you for your comments. The authors have added more detail. | Without theoretical guidance, important components can be omitted, and interventions may then fail to achieve the desired outcomes that create broader impact. Importantly, without theoretical explanation, it is not clear why interventions have succeeded or failed which prevents replication or duplication in other settings. | p.10 (p.9) |
| 1.15 | Pg8 – ‘To be effective interventions need to go beyond measuring the individual’s health determinants such as BMI or steps taken, number of fruit or vegetables consumed.’ – I do not understand what you are trying to say | Thank you for comment. We have revised the text to make the point clear. | Complex interventions need to measure social and economic health determinants, such as health equity51, access to healthy foods or safe exercise environments52, 53 to be able to capture social impact. | p.10 (p.9) |
| 1.16 | Pg8 – ‘The interventions which most clearly applied and measured social impact in children’s primary school health promotion were more likely to have used a theoretical lens’ – This should be in results section with more detail (eg., #s) | Thank you for the comment. We have added more detail to the results. | see Table 2). Outcomes of the interventions (the results or effects of a program and the changes that occur in attitudes, values, behaviours or conditions of interventions) were measured rather than the social impacts (the economic, social and environmental consequences, positive or negative, regardless of the purpose or perceived or real benefits of the activity)20 or theory used  …measurement was not methodical (assessing against a framework, theory or program logic) nor was it systematic (assessing all potential impacts – positive, negative intended or unintended) nor comprehensive (examining impacts in multiple domains such as individual, societal, economic and policy levels). | P. 7 (p.7)  p.7 (p.7) |
|  | **CONCLUSION** |  |  |  |
| 1.17 | P10 – ‘There is a need for social impact to be incorporated and evaluated in primary school health promotion interventions to increase the effectiveness, sustainability and accountability of interventions delivered in school settings’ – is there proof of this? | Thank you for your comment. We have revised the text to make our intended meaning clear. | There is a need for social impact to be incorporated and evaluated in primary school health promotion interventions to provide evidence of the benefits these interventions create and to demonstrate ‘value for money’. | p.12 (p.11) |
| 1.18 | ‘The first step is to develop a clear definition of what social impact is within health promotion.” – How is that the focus of the paper if there is no clear definition of it? | Thank you for the opportunity to respond to this. We have revised the text to draw a stronger conclusion. | This review indicated social impact measurement is poorly understood and measured Greater understanding is needed, and clear mapping of theory onto programs is required to explain why change occurs, and how this change leads to social impact. 65, 66 | p.12 (p.11-12) |
|  | **IMPLICATIONS** |  |  |  |
| 1.19 | Focus here seems to be on factors outside scope of study (social impact) and more broadly targeted to evaluating school-based health programs (theory, stakeholder engagement) | Thank you for your comment. We have addressed this as part of actions in response to other reviewer comments | Please refer to corrections: 1.6, 1.12, 1.13, 1.18, 1.21-1.23 |  |
| 1.20 | P10 - ‘This review sought to examine how social impact was measured within primary school health promotion interventions, and found significant gaps in how the longer terms effects of the intervention were considered or measured.’ – Did not mention longer term effects or really any particulars about the intervention effects in previous sections | Thank you for highlighting this inconsistency. We have altered this sentence to match the terminology used in previous sections of the paper. | Please refer to correction: 1.18 | p.12 (p.11-12) |
| 1.21 | P11 – ‘Evaluation needs to involve greater stakeholder engagement, and at a more in depth level, to establish and measure the longer term social impacts of an intervention, to understand if health promotion is effective within schools.’ – Many studies are likely prohibited in their ability (eg., cost) to study longer term outcomes; and again, not sure if this relationship was demonstrated through this study | Thank you for your feedback. We have revised the text to clarify these points. | Interventions need to target what matters to key stakeholders and encourage active participation if effective behavioural change is to be achieved.  Where possible, interventions need to plan for longer durations or frequent dosage. Stakeholder engagement and longer duration interventions are costly and resource intensive, and funding at this level not always available. However, to deliver broader social impact, and provide evidence that health promotion actions are value for money, these considerations are important. | p.13 (p.12) |
| 1.22 | P11 – ‘Moving forward, it is fundamental that interventions in primary schools consider: What impact should they see; what impact has occurred and the mechanisms; what types of impact have occurred; who has been affected or impacted, and to how to evidence impact for impact measurement models.’ - The article did not present literature supporting social impacts as fundamental | Thank you for the comment. We have deleted the word fundamental. | Moving forward, it is important that interventions in primary schools consider when measuring outcomes or social impact: | p.13 (p.13) |
|  | **OTHER** |  |  |  |
| 1.23 | Should the focus of discussion rather be on why so few studies measure social impact, what it is/how to define/measure it, the benefits of measuring it, possible barriers and how to increase the number of studies that do? | Thank you for the suggestion. | To advance effective health promotion, future research needs to address the barriers to implementing interventions which measure social impact. If we are to understand the value of measuring what has changed beyond the individual, clear mapping of the behavioural focus from input to outcome, stakeholder engagement and the measurement against theoretical constructs, needs to occur before a social impact chain can be established.’  Currently, the paucity of social impact research within this context of health promotion interventions limits understanding of the broader social, economic and health benefits of primary school health promotion, and social impact remains poorly defined. | In limitations p. 11 (p.11) |
| 1.24 | If only some of the studies had strong behavioral effects, can we realistically expect to see social impacts, particularly in those that had weak or no effects? | Thank you for the opportunity to address this comment. | If studies do not have strong behavioural effects, and create the predicted behavioural change, it is unlikely interventions will create social impacts, particularly for interventions which had weak or no effects. | p.9 (p.8) |
|  | **REFERENCES** |  |  |  |
|  | **TABLES** |  |  |  |

[1] Jurak G, Cooper A, Leskosek B, Kovac M. Long-term effects of a 4-year longitudinal school-based physical activity intervention on the physical fitness of children and youth during 7-year follow-up assessment. *Central European Journal of Public Health*. 2013; 21(4): 190-195.

[2] Ahrens W, Moreno LA, Mårild S, et al. Metabolic syndrome in young children: definitions and results of the IDEFICS study. *International Journal of Obesity*. 2014; 38(2): S4-S14.

[3] Rubinelli S, Diviani N. The bases of targeting behavior in health promotion and disease prevention. *Patient Education and Counseling*. 2020; 103(12): 2395-2399.

[4] Bandura A. Health promotion from the perspective of social cognitive theory. *Psychology & Health*. 1998; 13(4): 623-649.

[5] Barker F, Atkins L, de Lusignan S. Applying the COM-B behaviour model and behaviour change wheel to develop an intervention to improve hearing-aid use in adult auditory rehabilitation. *International Journal of Audiology*. 2016; 55(sup3): S90-S98.

[6] Howarth A, Quesada J, Silva J, Judycki S, Mills PR. The impact of digital health interventions on health-related outcomes in the workplace: A systematic review. *Digital Health*. 2018; 4.

[7] Rawhouser H, Cummings M, Newbert SL. Social impact measurement: Current approaches and future directions for social entrepreneurship research. *Entrepreneurship Theory and Practice*. 2019; 43(1): 82-115.
